# Serum metabolomics using ultra performance liquid chromatography coupled to mass spectrometry in lactating dairy cows following a single dose of sporidesmin

**DOI:** 10.1007/s11306-018-1358-4

**Published:** 2018-04-17

**Authors:** Zoe M. Matthews, Patrick J. B. Edwards, Ariane Kahnt, Mark G. Collett, Jonathan C. Marshall, Ashton C. Partridge, Scott J. Harrison, Karl Fraser, Mingshu Cao, Peter J. Derrick

**Affiliations:** 1 Massey University, Palmerston North, New Zealand; 2 University of Auckland, Auckland, New Zealand; 3 AgResearch Grasslands, Palmerston North, New Zealand

**Keywords:** Sporidesmin, Facial eczema, Mass spectrometry, Metabolomics, Bile acid, Dairy cow, Serum

## Abstract

**Introduction:**

Photosensitization is a common clinical sign in cows suffering from liver damage caused by the mycotoxin sporidesmin. This disease, called facial eczema (FE), is of major importance in New Zealand. Current techniques for diagnosing animals with subclinical sporidesmin-induced liver damage (i.e. without photosensitization) are nonspecific. In addition, little is known of the mechanisms involved in sporidesmin resistance, nor the early effects seen following low-dose sporidesmin intoxication.

**Objective:**

The objective of this study was to identify individual metabolites or metabolic profiles that could be used as serum markers for early stage FE in lactating cows.

**Methods:**

Results are presented from a 59-day sporidesmin challenge in Friesian-cross dairy cows. Serum metabolite profiles were obtained using reversed phase ultra-performance liquid chromatography (UPLC) electrospray ionization mass spectrometry (MS) and UPLC tandem MS. Multivariate and time series analyses were used to assess the data.

**Results:**

Statistical analysis, both with and without the temporal component, could distinguish the profiles of animals with clinical signs from the others, but not those affected subclinically. An increase in the concentrations of a combination of taurine- and glycine-conjugated secondary bile acids (BAs) was the most likely cause of the separation. This is the first time that MS methods have been applied to FE and that bile acids changes have been detected in cattle exposed to sporidesmin.

**Conclusions:**

It is well known that BA concentrations increase during cholestasis due to damage to bile ducts and leakage of the bile. This is the first study to investigate metabolomic changes in serum following a sporidesmin challenge. Further work to establish the significance of the elevation of individual BAs concentrations in the serum of early-stage sporidesmin-poisoned cows is necessary.

**Electronic supplementary material:**

The online version of this article (10.1007/s11306-018-1358-4) contains supplementary material, which is available to authorized users.

## Introduction

Sporidesmin, a mycotoxin produced by the saprophytic fungus, *Pithomyces chartarum*, is the cause of liver and bile duct damage that leads to elevated blood phytoporphyrin concentrations and a secondary photosensitization disease known as facial eczema (FE). Facial eczema predominantly affects sheep and cattle, and is of major economic importance in New Zealand (Smith and O’hara 1978; Di Menna et al. 2009).

Cows with clinical FE show reddening, swelling, and/or oozing and peeling of non-pigmented or sparsely haired skin, especially of the udder, teats, and escutcheon. In the early stages, behavioural observations can include shade-seeking, inappetence, and irritability or discomfort at milking. Besides the detrimental effects of FE on animal production and farm economics, the welfare of many photosensitized animals can be severely compromised (Morris et al. 2004).

Despite a large amount of FE research since the first report in sheep in 1897 (Lancashire and Keogh 1968) comparatively little has focused on the disease in cattle. Publications have concentrated on case reports from natural challenges, and on the genetic susceptibility of Friesian and Jersey cattle (Morris et al. 1991a, b, 1998, 2013). Furthermore, no data has been published on FE research using the modern techniques of metabolomics.

It has been estimated that for every animal that shows clinical signs, there are 10 that are subclinically affected (Morris 2011). Subclinically affected animals show no skin abnormalities, but liver damage can be revealed by blood biochemistry tests. Measurement of the serum activity of γ-glutamyltransferase (GGT) and glutamate dehydrogenase (GDH), enzymes which indicate bile duct and liver cell damage, respectively, have been used for many years to identify subclinical and clinical cases of FE (Morris et al. 2004, 2013; Phua et al. 2009). The activity of GGT, however, shows a lag phase of 10–14 days, before increasing (Bennison et al. 2010; Di Menna et al. 2009; Morris et al. 1998; Smith and Gravett 1986). An elevation of GGT activity may be relatively minor in subclinical cases, while animals that demonstrate elevations of a thousand units (IU/L) or more generally develop photosensitization (clinical FE). Although serum GGT testing is cheap and easy to perform, the interpretation of raised activities can provide false positives for FE, since other diseases, such as photosensitisation occurring on *Brassica* crops can cause similar enzyme elevations, clinical signs, and liver lesions (Collett 2014; Collett and Matthews 2014). Being able to identify whether a photosensitisation is caused by sporidesmin, or some other ‘toxin’, will allow for specific prevention, treatment and management methods, such as removing animals from toxic pastures, carrying out spore counting, or zinc treatment. Additionally, identifying the cause as brassica crops rather than FE will minimise additional environment stress caused by unneeded addition of toxic zinc treatments.

The response lag phase, and the lack of specificity, means that elevated GGT activity has limitations as a marker of sporidesmin toxicity.

With the aim to improve the diagnostic specificity of subclinical and clinical FE in cattle, we chose to apply modern analytical technologies [mass spectrometry (MS) and nuclear magnetic resonance (NMR)] in an attempt to discover one or more serum metabolite(s) that could be a diagnostic biomarker of sporidesmin exposure. Serum samples from cows that were non-responsive, that developed subclinical disease (as indicated by slight to moderate elevations in GGT activities), or that developed clinical photosensitivity (with highly elevated GGT and skin lesions), following a single dose of sporidesmin, were compared to normal, untreated control cows.

We report here the use of an untargeted approach using ultra performance liquid chromatography (UPLC) coupled to electrospray ionization MS (ESI-MS), followed by targeted analysis using a Tribrid MS, combining quadrupole, ion trap, and Orbitrap mass analyser (tandem MS or MS^2^). Such MS-based methods allow high selectivity, sensitivity and throughput, all ideally suited to the measurement of complex biological extracts (Cao et al. 2013).

In the present study, potential candidate markers, that included a combination of taurine- and glycine-conjugated secondary bile acids (BAs) were tentatively identified by accurate mass and by consulting online databases, such as the Human Metabolome Database (HMDB) (Wishart et al. 2013) and ChemSpider (Chemspider 2015), along with MS^2^ fragmentation data.

## Experimental

### Animals, dosing, and sample collection

All samples for this analysis were collected during a 59-day sporidesmin dosing trial carried out at Massey University, Palmerston North, New Zealand. All cows were subjected to a 14-day baseline control sampling period (Days − 14 to − 1) and 45 days of toxin response testing (Days 0–44). The study was approved by the Massey University Animal Ethics Committee (Protocol No. 11/23).

The sporidesmin was a crude extract obtained from spores of *P. chartarum* dissolved in 96% ethanol, prepared at AgResearch, Ruakura, Hamilton, New Zealand. Ethanol is the solvent routinely used in sporidesmin dosing studies (Amyes and Hawkes 2014). Seventeen of twenty adult Friesian and Friesian x Jersey, non-pregnant and lactating dairy cows, of mixed age, were treated with a sporidesmin dose (0.24 mg/kg; diluted with Milli-Q water) via intra-ruminal intubation, followed by 1 L of water. The remaining three cows served as controls and were subjected to the same regimen, excepting the sporidesmin dose was replaced by the equivalent volume of ethanol in water. Doses per cow, based on weight, are given in “Supplementary extended Appendix 1.”

Blood samples were collected into vacutainer tubes (no anticoagulant) every Monday, Wednesday, and Friday morning (*n* = 25 days). Two cows (239 and 393) were humanely killed before the trial was terminated due to the development of severe clinical FE, based on humane points for ethics, therefore provided only *n* = 15 samples/cow. All other cows had a total of *n* = 25 blood samples collected, per cow, for analytical chemistry analysis (total *n* = 480).

Once collected, blood samples were left to stand for ca. 30 min, and then centrifuged (3000 rpm, 6 min). The serum was transferred to cryovials and stored at − 80 °C pending analysis. An additional sample from each cow, collected every Monday, was submitted to New Zealand Veterinary Pathology (NZVP), Palmerston North, for serum biochemistry analysis (*n* = 172).

At the end of the sampling period, the treated cows were assigned to three groups based on their liver enzyme activities and photosensitisation observations. These groups were: non-responders (GGT activities within the reference range 0–36 IU/L for the duration of the trial), subclinical (elevated GGT activities, with no photosensitisation), and clinical (elevated GGT activities and photosensitisation).

### Liquid chromatography–mass spectrometry

Following mixing, 50 µL aliquots of thawed serum samples were treated with 220 µL of ice cold acetonitrile (LC–MS grade, Thermo Fisher Scientific, Auckland, NZ), vortexed, left to stand for 15 min and then centrifuged (13,000 rpm, 10 min). The supernatant was transferred to a microcentrifuge tube, and diluted with analytical grade water (Milli-Q® system, Millipore, Bedford, MA, USA), resulting in a total volume of 500 µL. Samples were mixed briefly (10 s vortex) and 200 µL was used for analysis by UPLC coupled to a high resolution Q Exactive Orbitrap mass spectrometer (Thermo Fisher Scientific, Waltham, MA, USA).

The analysis of all samples were performed using a C18 reversed-phase column (RRHD SB-C18 column, 2.1 mm × 150 mm, 1.8 µm diameter size, Agilent, CA, USA) based on the work by Fraser et al. (2013).

The UPLC system consisted of an Accela 1250 quaternary UPLC pump, a PAL autosampler fitted with a 15,000 psi injection valve (CTC Analytics AG., Zwingen, Switzerland), a 20 µL injection loop, and a Q Exactive Orbitrap mass spectrometer with electrospray ionization source (Thermo Fisher Scientific, Waltham, MA, USA). Please refer to ‘Supplementary experimental extended’ and Fraser et al. (2013) for detailed information on UPLC–MS experimental conditions.

Quality control samples, derived from a pooled 10 µL aliquot of all samples collected during the trial, were injected at the beginning, as every 10th injection thereafter, and at the end of each batch run. These control samples were formed to monitor systematic variations in run order and batch effects. A total of 690 samples were analysed in 7 batches.

### Tandem mass spectrometry coupled to liquid chromatography

Pooled serum samples were prepared by combining 100 µL of serum from each of three clinical cows (collected on Day 16 post-dosing). 1.2 mL acetonitrile was added to the combined sera, and vortexed. The solution was then centrifuged (12,000 rpm, 10 min, 4 °C). The supernatant was collected and dried in a centrifugal evaporator (Savant SC210A SpeedVac, Thermo Scientific). The dried residue was reconstituted in 100 µL of 50:50 (v/v) acetonitrile:water containing 0.1% formic acid.

The serum samples were analysed using a Dionex Ultimate 3000 UPLC system (Thermo Scientific), consisting of a pump, a column compartment (set to 30 °C), and an auto sampler (held at 10 °C). The chromatographic separation was carried out using two identical Acquity UPLC high strength silica (HSS) T3 columns (2.1 mm × 100 mm, particle size of 1.8 µm; Waters Corporation, Milford, MA, USA) connected in series to enhance separation.

An Orbitrap Fusion Tribrid mass spectrometer (Thermo Scientific) was used as the mass analyser, and was operated in both negative and positive ionisation modes.

Please refer to ‘Supplementary experimental extended’ for details of the experimental conditions used.

### Data processing and statistical analysis

To assess how the GGT activities changed over the trial period for each animal, and each group, a generalised additive model (GAM) (Hastie and Tibshirani 1990) was fitted to the data, using RStudio software (Version 0.97.449, RStudio, Boston, MA, USA), allowing for each group to have a separate smooth average, taking into account the initial weight of the animals. Typically, the activity of GGT following sporidesmin challenge was skewed, since individual cows, within a group, could exhibit highly variable levels of response, in which case, natural log (ln) transformed data were used.

For the UPLC/MS, the components with a retention time (RT) before 3 min, and after 14 min, were considered as waste; the remaining data were extracted and aligned using in-house proprietary software developed at AgResearch, Palmerston North, NZ.

Raw data was subjected to a peak detection process using PhenoAnalyzer (SpectralWorks Ltd, Manchester, UK). The key parameter settings were: area threshold = 100,000; peak width threshold = 0.06 min minimum width to 0.4 min maximum width; *m*/z peak detection window = 10 ppm (Fraser et al. 2014). The resulting peak-area matrix data were de-isotoped using a procedure described previously (Cao et al. 2017). Any isotopes not removed by the automated de-isotoping process were removed during data processing. Run-order effects within each batch were normalized using linear regression (Koulman et al. 2007) and the batch effect was corrected using a parametric empirical Bayes method (sva package, RStudio) (Johnson et al. 2007). Furthermore, the peaks still showing a significant batch effect (*F* test, *p* values < 0.05) were removed, as described by Cao et al. (2017). Following the sva correction, 42 peaks were removed from the CP dataset and 7 from the CN dataset (see ‘Supplementary extended Appendix 3’ for a list of removed peaks). The data were ln-transformed prior to multivariate analysis (MVA) as they did not follow a normal distribution.

Following pre-treatment, a total of 450 peaks (*m*/*z*_RT) were detected within the (−) ESI (CN) chromatograms and 1278 peaks (*m*/*z*_RT) in the (+) ESI (CP) chromatograms. Matrix tables were produced with the predictor variables (*m*/*z*_RT values) in the *X*-block and the response variables (group, day, dosing) in the *Y*-block. The resulting matrices were then analysed using SIMCA (Version 13.0.2.0, Umetrics AB, Sweden) and RStudio statistical software.

Principal component analysis (PCA), partial least squares-discriminate analysis (PLS-DA), and orthogonal PLS-DA (OPLS-DA) were performed using SIMCA. All data were pre-treated using Pareto scaling.

PLS-DA and OPLS-DA models were produced using defined classes to aid in separation of the data based on specific observations. For example, samples were classed by those measured before dosing (Days − 14 to 0) compared to those measured after dosing (Days 7–42). All PLS-DA models were validated using permutation testing (999 permutations). Further, a reliable, validated model was considered to be one with: (a) a Variable Influence of Projection (VIPcv) score of > 1, (b) a correlation (p(corr)[1]) generally > 0.4, and (c) loadings columns with jack-knifed confidence intervals not crossing the origin. Lists of the peaks (*m*/*z*_RT) of likely candidates for the separation of classes produced from this analysis were used for data interpretation.

Time series analyses were applied to the data to explore whether any mz_RT peaks may have differed over time, and between groups over time. Time series analyses were performed using in-house routines written for RStudio. *m*/*z*_RT peak intensity versus time curves were plotted for each cow. The m/z_RT peaks that differed the most between groups were identified by ranking using two techniques. The first was shrinkage discriminant analysis (SDA) which computes correlation-adjusted T-scores (CAT) between the group centroids and the pooled mean for each peak (Ahdesmaki and Strimmer 2010). The second was a permutation-based *p* value, where the ratio of between and within group variance was computed for each peak. The groups were then permuted and the ratio recomputed. The *p*-value was then calculated as the proportion of permuted ratios that were larger than the ratio from the observed data, with a small *p* value suggesting that the results identified in the data were unlikely to have arisen by chance. These *p* values were not corrected for multiple testing, thus a *p* value < 0.05 was not regarded as statistically significant. However, ranking by *p* value did allow the identification of peaks that were more likely to separate the groups. Full details of all of the statistical methods are given in the ‘supplementary experimental extended: Methods.’

Once important *m*/*z_*RT pairs were identified for both the MVA and the time series models, the raw spectra were examined to determine whether these variables related to ‘true’ peaks, rather than noise, whether they were well resolved, and had high signal to noise ratios (S/N > 3). Once the peaks fulfilled the criteria, a combination of metabolite databases: the HMDB (Wishart et al. 2013), ChemSpider (Chemspider 2015), published data (Allen et al. 2011; Griffiths and Sjovall 2010), and MS^2^ fragmentations were used to tentatively identify metabolites.

## Results

### Serum biochemistry

Of the 17 treated cows, four were classified as non-responders, six subclinical, and seven as clinical (Table 1). The first clinical cases (*n* = *2*) occurred on Day 7 and Day 9 following sporidesmin dosing.

**Table 1:** Categorization of groups based on elevated liver enzyme activities following sporidesmin dosing and visual signs of photosensitivity

Category	n	Cow ID	GGT* (IU/L 37 °C)	GDH** (IU/L 37 °C)	Clinical signs
Normal					
Control	3	22, 152, 384	21 (16–27)	15 (7–59)	None
Non-responder	4	195, 244, 374, 448	21 (13–33)	18 (3–65)	None
Responders					
Subclinical	6	64, 222, 312, 395, 420, 424	56 (15–288)	44 (6–389)	None
Clinical	7	239, 282, 298, 317, 393, 440, 450	1102 (116–2377)	440 (11–2420)	Abnormal irritability, shade seeking, increased sensitivity, reddening of non-pigmented skin, peeling of skin***

Measured GGT and GDH activities are presented as median (range) for the group from the trial, after Day 0

* γ-Glutamyl transferase; normal range is 0–36 UI/L at 37 °C for New Zealand Veterinary Pathology laboratories

** Glutamate dehydrogenase; normal range is 8–41 UI/L at 37 °C for New Zealand Veterinary Pathology laboratories

*** The first clinical signs appeared at Day 7, with 393 having increased sensitivity and irritability in the milking shed. The remaining cows began to show signs around Day 9 (shade seeking and/or reddening of teats)

There was no correlation between the time when clinical photosensitivity became evident and the maximum elevation of GGT. For example, one cow had a GGT activity of 863 IU/L three days prior, while a second cow had a GGT of 116 IU/L two days prior to the signs emerging.

**Fig. 1:**
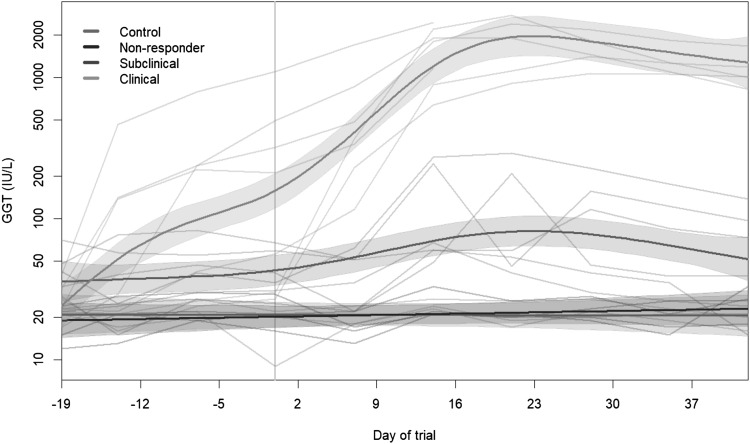
γ-Glutamyltransferase (GGT) activities for each of the four defined groups, with within group averages fit, calculated using a generalised additive model. log_e_ transformed data was used. Day 0 refers to the day of dosing. Shaded areas represent the 95% confidence bands for that group

In Fig. 1 changes in GGT activities for both the individual cows as well as the average within the groups, across time, are shown. The GGT activities remained well within the normal range for controls and non-responders, while they increased between Days 7 and 14 for subclinical animals, followed by recovery after around Day 21. No differences were identified between control cows and non-responders over the trial period. However, clinical animals that had elevated levels through the entire trial exhibit an increase in liver enzyme activities well above normal, following dosing (Day 0). There is a clear difference between groups (p < 0.0001) when compared to a baseline model with a common trend for all groups and this is primarily due to the clinical group differing (Fig. 1) from other groups. Within the clinical cow group, cows 282 and 393 exhibited more severe responses to the sporidesmin dosing than all other cows. To ensure these cows did not have a strong effect on the mean-based measures used in GAM, samples from cows 282 and 393 were removed and the models re-run (See supplementary extended Appendix 2 for plots produced from these models). When this model was compared to the original model with all 20 cows, the results remained the same (p < 0.0001) (Figures a and b in Supplementary extended Appendix 2) therefore all cows were retained in the dataset.

### Multivariate analysis

**Fig. 2:**
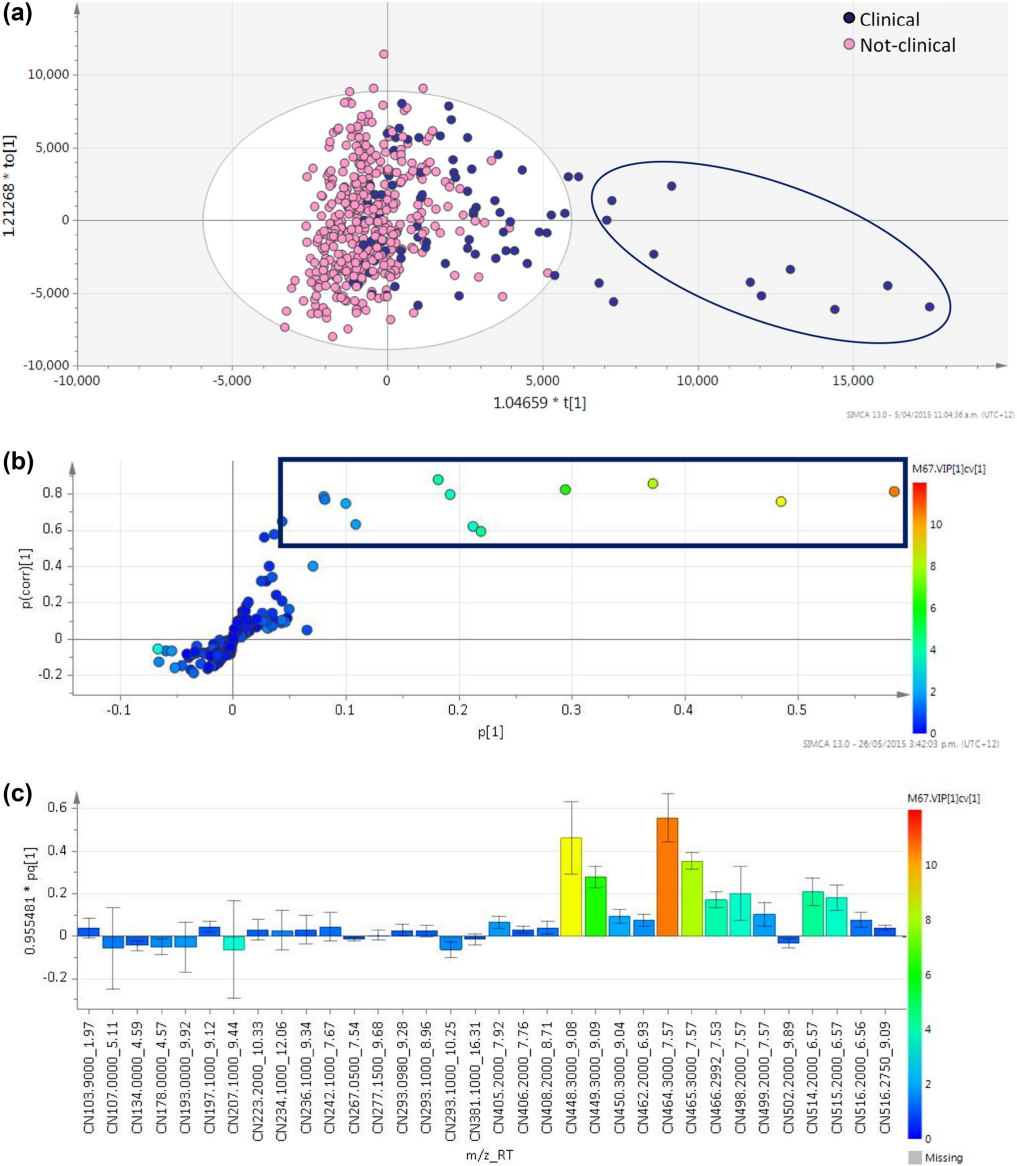
CN derived data showing orthogonal partial least squares-discriminant analysis (OPLS-DA) classed by clinical cows versus all other cows, using Pareto scaling; **a** Scores plot coloured by classes. Cow 282 clustering is shown by the ellipse; **b** S-plot, coloured by Variable Influence of Projection (VIPcv) (box = p(corr)[1] > 0.5; VIPcv > 1); and **c** loadings column plot showing all those with a VIPcv > 1

**Fig. 3:**
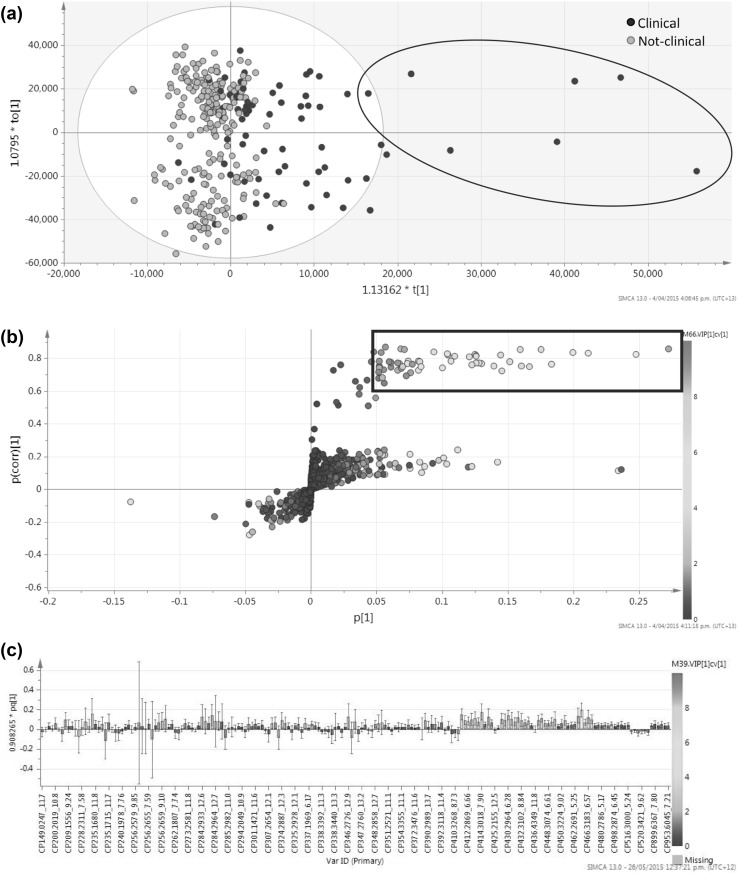
CP derived data, showing orthogonal partial least squares discriminant analysis (OPLS-DA), classed by the clinical cow group, after dosing, versus all other cows and control days, using Pareto scaling; **a** scores plot, coloured by classes. Cow 282 clustering is represented by the ellipse; **b** S-plot, coloured by Variable influence of Projection (VIPcv) (box shows p(corr)[1] > 0.5 and VIPcv > 1); and **c** loadings column plot showing all m/z with a VIPcv > 1

Both the CN and CP datasets showed significant differences (CN *p* = 1.88 × 10^− 28^, CP *p* = 4.63 × 10^− 9^, OPLS-DA) between clinical cattle after dosing, and all other animal groups (Figs. 2, 3), with the clinical cattle moving away from the other cow groups following a trend with time after sporidesmin dosing, and returning towards the other groups in the later weeks of the trial, consistent with a recovery phase.

The PLS-DA models were validated, with a difference between the goodness of fit (R^2^ 0.298 and 0.335) and the predictive ability (Q^2^ 0.201 and 0.249) of 0.097 and 0.086 for the CP and CN datasets, respectively. The R^2^/Q^2^ values were themselves low, and not considered reliable indicators by SIMCA definition; however, because of the inherent variable nature of large metabolomic datasets of biological origin, and the closeness of these two attributes to each other, these models were accepted (Worley and Powers 2013). In the scores plots (Figs. 2a, 3a), those samples sitting outside the grey confidence ellipse (T^2^ > 95%), were not considered to be outliers, as they related to weeks 4, 5, and 6 of the trial (Days 7, 9, 11, 14, 16, and 21)—the earlier days representing a change from ‘normal’ after dosing, and the latter (being closer to zero on the *x* axis), representing a return to likeness of the other samples. Furthermore, most of the individual clinical cows appeared to cluster together, whereas cow 282, a clinical cow, differentiated the most from all, and was seen to orientate in the bottom right of the OPLS-DA plots (blue ellipse, Figs. 2a, 3a). Cow 282 was one of the more severely affected cows in the trial. In addition, these were the weeks where all of the clinical cattle had their highest GGT activity and began showing clinical signs.

The S-plot combined with VIPcv colouring (VIPcv > 1) and jack-knifed confidence levels (> 0) indicated 22 peaks and 76 *m*/*z*_RT peaks which were relevant to class separation from the CN and CP datasets respectively. Of the 22 peaks from the CN dataset, a total of 9 up-regulated peaks were identified as being reliably relevant to class separation. From these, the ions at *m*/z 464.3017 at 7.57 min and 448.3074 at 9.08 min were major contributors to the differentiation between the classes. These two ions were observed as the most important in the differentiation of clinical cows from all others after dosing, with considerably higher peak areas in the first two weeks after dosing, than in all other samples. These ions could be tentatively identified using accurate mass and the HMDB as glycocholic acid and glycochenodeoxycholic acid.

Of the 76 peaks in the CP dataset, 57 had a p(corr)[1] > 0.5. These 57 up-regulated peaks were considered reliable for class separation. Of these, the 6 major contributors to the class separation were at *m*/*z* 466.3174 at 6.32 min, 414.3018 at 7.90 min, 412.2866 at 6.38 min, 430.2964 at 6.28 min, 466.3155 at 7.31 min, and 448.3075 at 6.31 min (Supplementary extended Appendix 4).

Accurate masses were used to tentatively identify the metabolites using the HMDB. These m/z values were consistent with a combination of BAs, such as glycocholic acid and 3-oxo-4,6, choladienoic acid, as well as amino acid derivatives, and minor metabolites of fatty acids. A number of potential isobaric isomers were also detected, including *m*/z 337.2529 (RT 6.31) and 337.2540 (RT 6.53) ([M + H]^+^).

Table 2 and supplementary extended Appendix 4 summarise the peaks that were responsible for the differences between the clinical cattle compared to the other groups, as observed in the OPLS-DA models for CN and CP data, respectively.

**Table 2:** Summary of detected compounds from CN LC–MS and MS^2^ analysis of serum from the clinical cows, using MVA and time series techniques

[M–H]^−^ *m*/*z*	LC–MS RT (min)	[M–H]^−^ *m*/*z*	LC–MS/MS RT (min)	MS^2^ product ions (abundance) [loss (amu)]	Elemental composition of the parent ion	Error (ppm)	Proposed metabolite identity
462.28673	6.93	462.29218	8.53	444.27602 (30%) (− 18) 418.29699 (100%) (− 43) 400.28595 (20%) (− 62)	C_26_H_40_O_6_N C_27_H_36_O_2_N_5_ C_13_H_38_O_8_N_10_	3.646 0.818 − 0.280	Unknown
464.30175	7.57	464.30758	9.12	446.29515 (20%) (− 18) 420.31569 (20%) (− 43) 402.30485 (100%) (− 62)	C_26_H_42_O_6_N	− 0.024	Glycocholic acid
464.30329	6.56	464.30692	9.12	446.29279 (20%) (− 18) 420.31555 (25%) (− 44) 402.30515 (100%) (− 62) 384.29175 (14%) (− 80) 353.25220 (5%) (− 111) 400.28992 (10%) (− 64)	C_26_H_42_O_6_N	3.4	Glycocholic acid
516.27500	9.09	516.29710	10.60	448.30762 (100%) (− 67)	Unknown	–	Unknown
514.28452	6.57	514.29800	8.89	496.27597 (10%) (− 18) 412.28754 (5%) (− 102) 371.26074 (5%) (− 143) 353.25009 (8%) (− 161)	C_26_H_45_O_7_NS	0.123	Taurocholic acid
478.31760	7.57	478.29720	13.07	281.25035 (100%) (− 197) 214.04946 (2%) (− 264) 196.03867 (4%) (− 282)	Unknown	–	Unknown
448.30746	9.08	448.31360	10.60	430.29821 (15%) (− 18) 404.31877 (100%) (− 44) 386.30826 (5%) (− 62) 355.26639 (5%) (− 93)	C_26_H_42_O_5_N	1.368	Glycochenodeoxycholic acid / Deoxyglycocholic acid
432.31274	10.94	432.31779	12.18	388.32431 (100%) (− 44) 173.48961 (15%) (− 275)	C_26_H_42_O_4_N	1.869	Lithocholic acid glycine conjugate
498.29104	7.57	498.29105	10.74	355.26587 (100%) (− 143) 414.30334 (5%) (− 84)	C_26_H_45_NO_6_S	3.127	Taurochenodeoxycholic acid/Taurodeoxycholic acid

All components were up-regulated in comparison to the other cow groups

No validated models could be used to separate any other combinations of the treated groups.

### Time series analysis

**Fig. 4:**
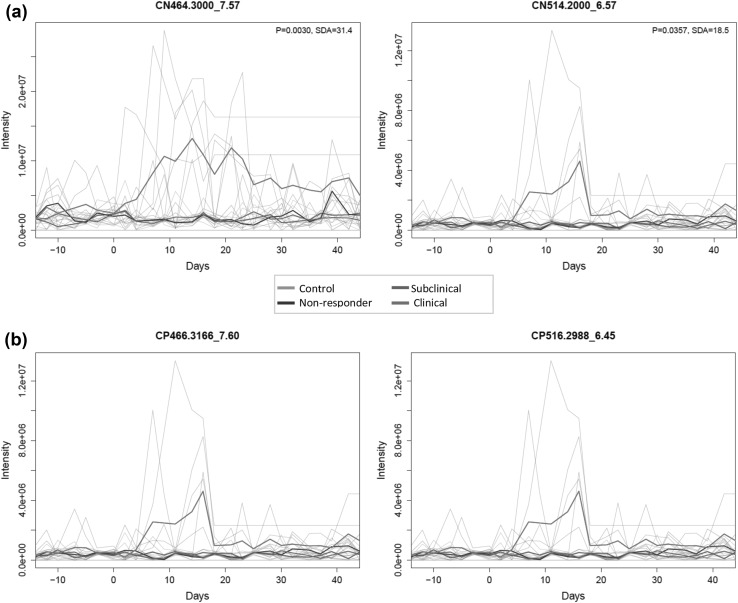
Time series of the main two mz_RT peaks identified as showing a major difference in the clinical cows, only after dosing (Day 0; p < 0.05 and true peaks), derived from **a** CN, and **b** CP ESI-MS analysis. The compounds were shown to increase from ca. Day 5 onwards, before any significant increases in GGT activities were observed. The observed temporal variations, and corresponding RT and m/z values, could be attributed to equivalent compounds, i.e. MW 465 (left) and MW 515 (right). Pale lines represent the time profiles of each m/z_RT pair for each cow. The thick lines represent the average time profiles for the groups

Changes in the amount of certain metabolites detected in both CN and CP modes were seen between control cows and the clinical cows after dosing. The changes in the curves follow an expected shape, increasing after dosing and decreasing at the end of the trial. The exclusion of the clinical cow group revealed no obvious differences between the remaining groups (controls, non-responders, and subclinicals). The peaks in the raw LC–MS spectra that showed a clear visual group differentiation were examined in order to detect false positives and it was noted whether any compounds were detected in both the CN and CP ionization modes. Figure 4 shows an example of this, where corresponding *m*/z values were detected.

In summary, nine and twenty-two peaks that were considered to be the main components causing the observed differences between clinical and all other cow groups were extracted from both the CN and the CP datasets (summarised in Table 2 and supplementary extended Appendix 4, respectively). Of the peaks identified as major contributors to the differentiation of clinical cows by the time series methods, five of the nine CN peaks and 18 of the 22 CP peaks were also identified by OPLS-DA. This shows the complementary nature of these two techniques.

### Targeted analysis using the Orbitrap tandem mass spectrometer

According to VIPcv values, time series combined rankings, and the examination of raw spectra, a total of nine and 22 peaks from CN and CP ESI-MS analysis, respectively, were selected for MS^2^ analysis (Table 2 and supplementary extended Appendix 4, respectively).

**Fig. 5:**
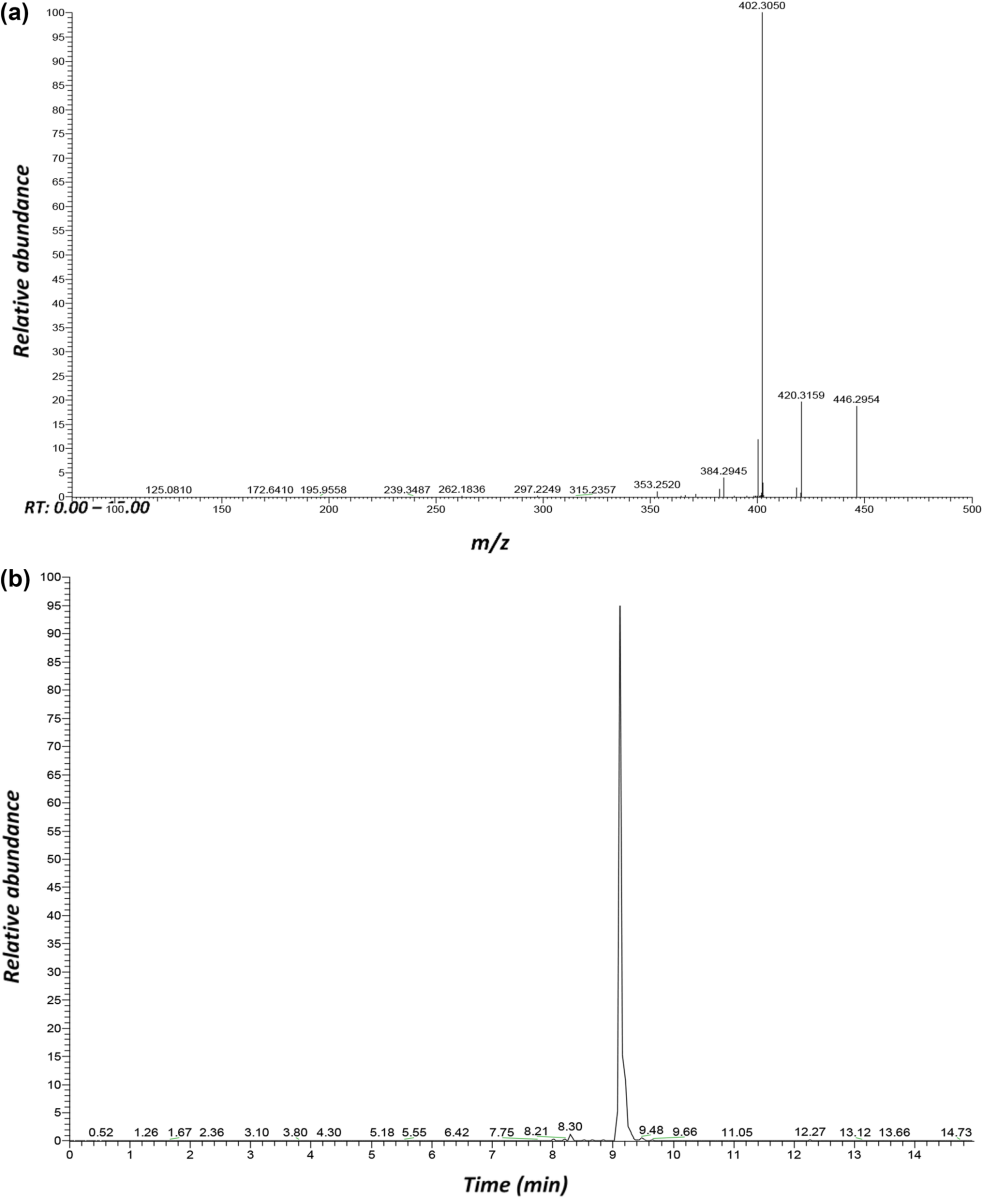
Liquid chromatography negative electrospray ionisation tandem mass spectrometry analysis of serum, showing the m/z 464 MS2 data (**a**) eluting at 9.12 min (**b**)

As an example from the CN dataset, chromatographic and MS^2^ data are shown in Fig. 5 for the compound eluting at 9.12 min in the pooled sample. For *m*/z 464, the product ions at *m*/z 420.3159 (loss of 44 amu, 1×CO_2_), 446.2954 (loss of 18 amu, 1×H_2_O), and 402.3050 (loss of 62 amu, H_2_O + CO_2_) were formed during the MS^2^ analysis, tentatively indicating that it is a type of organic acid.

A corresponding protonated compound ([M + H]^+^) was detected at *m*/z 466 at 9.15 min from the CP analysis. The product ions at *m*/*z* 448.3046, 430.2940, and 412.2833 were observed during the MS^2^ analysis. Owing to the detection from both MS and MS^2^ analysis, the ions 448.3046 (loss of 18 amu, 1 × H_2_O), 430.2940 (loss of 36 amu, 2 × H_2_0), and 412.2833 (loss of 54 amu, 3 × H_2_O) originate from the same compound *m*/z 466.3166, which readily loses water in CP-MS analysis. Neutral water loss is common for hydroxylated compounds, and the occurrence of multiple water losses lead to the conclusion that *m*/*z* 466.3166 contains three hydroxyl groups. An elemental composition of C_26_H_44_NO_6_^+^ (0.612 ppm error) was determined. Taking into account that the molecule contains three OH groups, it is most likely glycocholic acid. For the *m*/*z* 464.3018 (RT 9.12, [M–H]^−^) a likely elemental composition of C_26_H_42_NO_6_^−^ (− 0.024 ppm error) was also determined, and is also likely to be glycocholic acid detected in negative ionization mode. Another *m*/*z* 464.3033 (RT 6.56) peak was identified as being instrumental in the difference between clinical and all other cows after dosing. With similar accurate masses and product ions, it is likely that these are isobaric isomers. Other isobaric isomers were detected, including *m*/*z* 472.3031 (RT 8.78) and 472.3039 (RT 7.66) in the CP MS spectra. The protonated compound ([M + H]^+^) detected at *m*/z 450.3209 at 8.52 min from the CP-MS analysis produced two main product ions, 432.3087 and 414.2982. The fragmentation pattern, showing a loss of one and two H_2_O molecules, matched with that of glycochenodeoxycholic acid (− 3.546 and 0.228 ppm errors, respectively). Accurate mass measurements for both the precursor and product ions are summarized in Table 2 and supplementary extended Appendix 4 from the CN and CP MS^2^ analysis, respectively.

The most prominent changes seen between the groups, after dosing, were observable among the compound class of BAs. Distinguishing between the different BAs is often difficult, with the only differences being the presence or absence of hydroxyl groups in positions 3, 7, and 12. Tentatively, five BAs were detected that exhibited an increase in the clinical group after dosing, but not in the other groups. Glycocholic acid, taurocholic acid, taurochenodeoxycholic acid, and glycochenodeoxycholic acid (or deoxycholic acid) were detected in both CP and CN datasets, providing strong evidence for their presence in clinical cattle following liver damage caused by a sporidesmin dose. Lithocholic acid was only detected in the CN dataset.

## Discussion

The intention of this work was to differentiate between cows responding variably to a single sporidesmin dose, using UPLC/ESI-MS and UPLC/MS^2^ of serum, with the aim to use individual metabolites as potential serum markers for early stage FE in lactating cows. The clinical cow group was able to be clearly differentiated using chromatography coupled to CP and CN ESI-MS. Tandem MS was able to tentatively identify nine of the peaks that were relevant to clinical cows after dosing. All of the identified peaks were taurine- and glycine-conjugated secondary BAs. Bile acids are physiological detergents that facilitate excretion, absorption, and transport of fats and sterols in the intestine and liver, and are essential for the digestion and absorption of hydrophobic nutrients (Griffiths and Sjovall 2010; Chiang 2009).

For the clinical cow group, glycocholic acid, taurocholic acid, taurochenodeoxycholic acid, as well as one peak that was narrowed down to two likely BAs, glycochenodeoxycholic acid or deoxyglycocholic acid, appeared in both CN and CP datasets as being elevated. A lithocholic acid-glycine conjugate was identified in negative mode only. All of these metabolites were elevated in the clinical cows, when compared to the control days for the same cows, plus the control cows, and all other groups after dosing. The weeks where the concentrations of BAs were elevated in these cows correlated with the highest GGT activity and when these cows started showing clinical signs of FE. The pattern of an increase directly after toxin ingestion followed by a decrease can be expected with disease progression. This clearly correlated with the time-series analysis where an increase in BAs is observed from day 5 onwards (post-dose) then begin returning to normal towards the end of the trial..

Three of the seven cows categorised as clinical, based on liver enzyme activities and clinical observations, showed elevations in GGT activities before the dose of sporidesmin was administered on Day 0. The reason for this is unknown. The cows originated from the same farm which had no history of FE, and prior to purchase the GGT activities were all within normal reference range. On arrival at Massey, all cows were grazed on paddocks that had been sprayed with a fungicide and *P. charatarum* spore counts in pasture and faeces had been assessed before and during the trial. The three control cows were randomly selected, and all cows received the same dose for their body weight. In addition, the livers of all cows were examined grossly and histologically, and none showed any evidence of chronic (i.e. pre-existing) sporidesmin damage. Therefore, we concluded that the elevations in enzyme activities before dosing was not related to sporidesmin exposure at the farm of origin or at Massey University.

In addition, although there are other mycotoxins which are commonly present in pastures around the same time as sporidesmin, such as ryegrass and fescue endophytes (Nicol and Klotz 2016; Rogers et al. 2016), the compounds produced by these endophytes do not cause liver damage (Rogers et al. 2016; Nicol and Klotz 2016; Di Menna et al. 2012). Aflatoxin, which is also present when humid conditions develop, does cause liver damage, however is a storage mycotoxin and therefore present in post-harvest stored grains nuts (Keyl and Booth 1971), which the cattle in this trial had no access to. The liver lesions cause by aflatoxin, are distinctly different to those caused by sporidesmin (Cullen and Stalker 2016), and were not detected during histological analysis.

It is well established that concentrations of BAs increase in the liver and blood during cholestasis due to leakage of bile into the parenchyma of the liver (Woolbright et al. 2014; Zhang et al. 2012; Fickert et al. 2002). It is often assumed that the increases in cytotoxic BAs are the cause of, or at least involved in, the progressive hepatocellular injury that occurs during cholestasis. Zhang et al. (2012) showed that the concentration of BAs was elevated in the serum after bile duct ligation in rats. Chenodeoxycholate is known to be directly cytotoxic for hepatocytes (Miyazaki et al. 1984). In addition, lithocholic acid, a bacterial metabolite of chenodeoxycholate produced in the intestinal tract, has been implicated as a possible hepatotoxin (Miyazaki et al. 1984). Glycochenodeoxycholic acid is the most toxic known form of chenodeoxycholate, and has also been shown to induce hepatocellular injury in a dose-dependent manner in human patients (Spivey et al. 1993; Trottier et al. 2012; Woolbright et al. 2015; Siviero et al. 2008).

Most studies focus on glycochenodeoxycholic acid and taurocholic acid, with some mention of taurochenodeoxycholic acid, glycocholic acid, lithocholic acid, and deoxyglycocholic acid. One study showed that 95% of biliary BAs in bovids was made up of three types: cholic acid, chenodeoxycholic acid and deoxycholic acid (Hagey et al. 1997). It has been suggested that glycine-conjugated BAs are more cytotoxic than those that are taurine-conjugated; however this may be due to the greater prevalence of glycine-conjugated BAs (Woolbright et al. 2015). Both glycine- and taurine-conjugated BAs were identified in the serum of clinical cows in the present study.

Other recent studies have suggested that BAs are not directly hepatotoxic, but instead increase the expression of proinflammatory mediators, such as cytokines, that promote hepatic inflammation and significant liver injury through the recruitment of neutrophils and macrophages (Allen et al. 2011; Woolbright et al. 2015; Zhang et al. 2012).

Irrespective of the mechanism, it appears that the increase in the concentration of various serum BAs seen in cows that became clinically affected, occurs following primary damage to bile ducts. Sporidesmin causes a necrotising cholangitis (Hohenboken et al. 2000), which manifests as cholestasis in severe cases. In addition to cholestasis, sporidesmin has been shown to inhibit BA uptake into hepatocytes (Cordiner and Jordan 1983). Furthermore, previous work by Gallagher (1964) and Middleton (1974) demonstrated sporidesmin-induced mitochondrial swelling, suggesting that sporidesmin acts on hepatocyte membranes, and may increase permeability, and efflux of BAs from the cytoplasm.

In conclusion, the use of MS provided additional data concerning the latter stages of clinical FE in dairy cattle. Sporidesmin damage, at the dose given in this study, appeared to manifest itself quickly in those cows that were more susceptible to the toxin. It is hypothesised that any metabolites produced directly from sporidesmin, and/or other compounds, during the initial attack on the bile ducts would have been removed from the blood before the first blood samples were taken, 48 h after dosing. The results presented here could be used as a basis for more targeted analysis in the immediate aftermath of dosing, better processing, refinement of equipment parameters, accurate quantification, and identification of marker metabolites. To our knowledge this is the first time an elevation of deoxyglycholic acid has been detected in association with bovine FE. Future studies should investigate the BAs as a target set of biomarkers for liver damage caused by sporidesmin intoxication.

## Electronic supplementary material

Below is the link to the electronic supplementary material.

Supplementary material 1 (DOCX 124 KB)

Supplementary material 2 (DOCX 26 KB)
