# Supplementary material for: Serum metabolomics using ultra performance liquid chromatography coupled to mass spectrometry in lactating dairy cows following a single dose of sporidesmin
Source: Metabolomics. 2018 Apr 17;14(5):61. doi: 10.1007/s11306-018-1358-4 (PMC5904237; doi:10.1007/s11306-018-1358-4)
Supplement: Supplementary file 1 — Supplementary material 1 (DOCX 124 KB) [file 11306_2018_1358_MOESM1_ESM.docx]

# Supplementary extended: Appendix

# Journal: Metabolomics

# Serum metabolomics using ultra performance liquid chromatography coupled to mass spectrometry in lactating dairy cows following a single dose of sporidesmin

## Zoe M Matthews^1^, Patrick J B Edwards^1^, Ariane Kahnt^2^, Mark G Collett^1^, Jonathan C Marshall ^1^, Ashton C Partridge^2^, Scott J Harrison^2^, Karl Fraser^3^, Mingshu Cao^3,^ Peter J Derrick^2*^

^1^ Massey University, Palmerston North, New Zealand; ^2^  University of Auckland, Auckland, New Zealand; ^3^ AgResearch Grasslands, Palmerston North, New Zealand,
* Deceased.

^§^ Authors for correspondence: [z.matthews@massey.ac.nz](mailto:z.matthews@massey.ac.nz)
 [p.j.edwards@massey.ac.nz](mailto:p.j.edwards@massey.ac.nz)

***Appendix 1*** The sporidesmin doses administered on Day 0. The sporidesmin in ethanol dose was per animal based on its weight on arrival on 30.03.2011 (Day - 19), and diluted 1:10 with Milli-Q water.

| **ID No.** | **Weight (kg) (30.03.2011)** | **Sporidesmin dose (mg)** | **Ethanol control dose (mg)** | **Group** |
| --- | --- | --- | --- | --- |
| 22* | 432 | - | 103.68 | Control |
| 64 | 387 | 92.88 | - | Treated |
| 152* | 445 | - | 106.80 | Control |
| 195 | 494 | 118.56 | - | Treated |
| 222 | 388 | 93.12 | - | Treated |
| 239 | 395 | 94.80 | - | Treated |
| 244 | 502 | 120.48 | - | Treated |
| 282 | 403 | 96.72 | - | Treated |
| 298 | 426 | 102.24 | - | Treated |
| 312 | 405 | 97.20 | - | Treated |
| 317 | 413 | 99.12 | - | Treated |
| 374 | 468 | 112.32 | - | Treated |
| 384* | 464 | - | 111.36 | Control |
| 393 | 396 | 95.04 | - | Treated |
| 395 | 430 | 103.20 | - | Treated |
| 420 | 548 | 131.52 | - | Treated |
| 424 | 410 | 98.40 | - | Treated |
| 440 | 397 | 95.28 | - | Treated |
| 448 | 495 | 118.8 | - | Treated |
| 450 | 526 | 126.24 | - | Treated |

*Control cows were given a control dose of ethanol, based on weight, diluted with Milli-Q water. Volume of ethanol calculated for 96% ethanol, density of 0.789 g/ml.

***Appendix 2 Gam modelling of GGT*** – exclusion of outliers to determine if there is an effect on the mean-based modelling.

**Figure a** ɤ-Glutamyltransferase (GGT) activities for each of the four defined groups, excluding cow 282, showing the difference between groups (*p* < 0.0001), calculated using a generalised additive model. log_e_ transformed data was used. Day 0 refers to the day of dosing. Shaded areas represent the 95% confidence bands for that group.

**
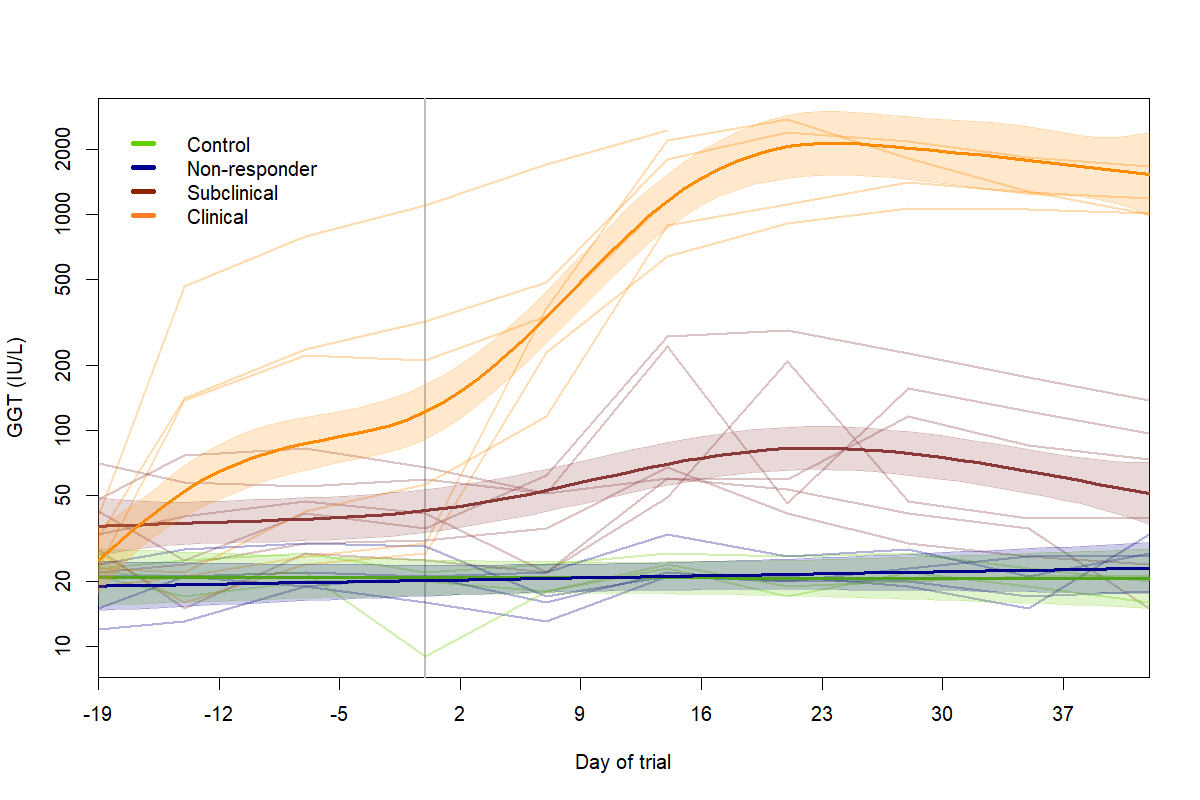
**

**Figure b** ɤ-Glutamyltransferase (GGT) activities for each of the four defined groups, excluding cow 282 and 393, showing the difference between groups (*p* < 0.0001), calculated using a generalised additive model. log_e_ transformed data was used. Day 0 refers to the day of dosing. Shaded areas represent the 95% confidence bands for that group.

**
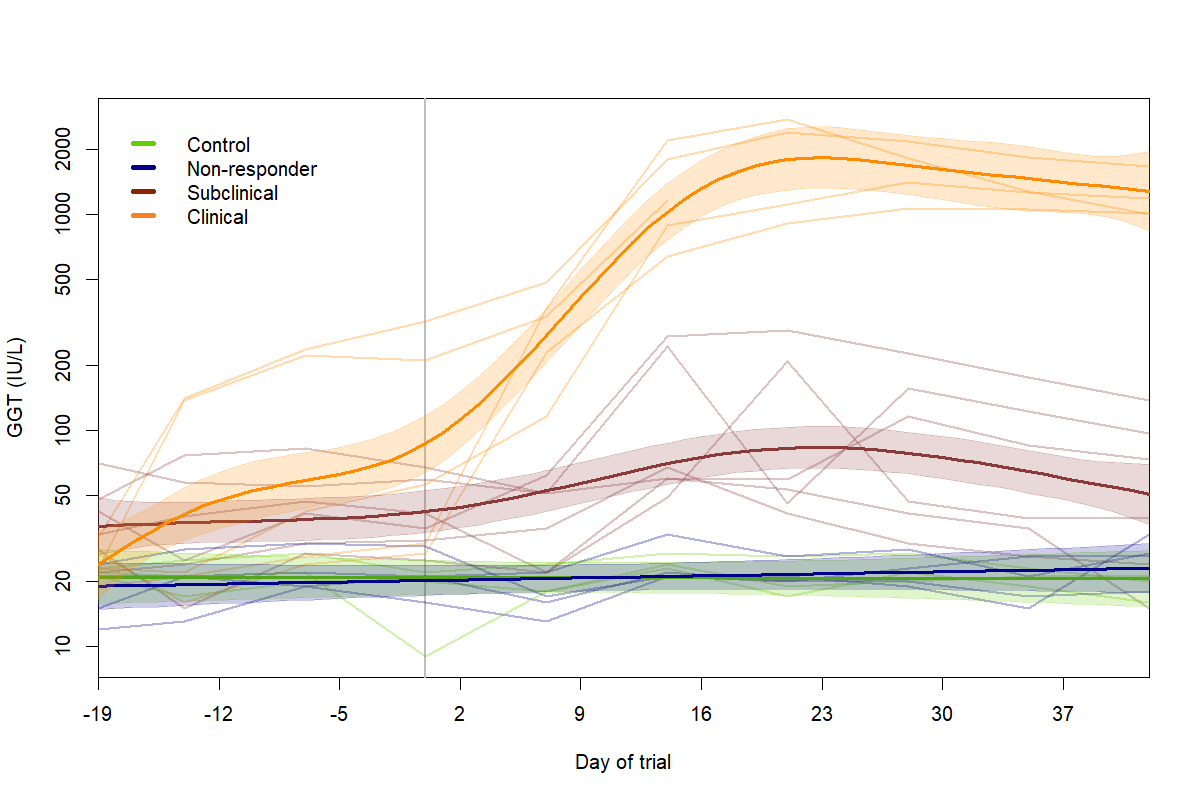
**

***Appendix 3 C18 positive and negative:*** peaks excluded following sva batch correction and isotopic peak identification

**CP data**42 peaks were removed following sva batch correction

[1] "CP84.0656_0.93" "CP95.0699_7.44" "CP210.1205_7.97" "CP216.1564_3.33"

[5] "CP226.1399_6.19" "CP229.1803_7.56" "CP229.1987_8.53" "CP250.1343_7.41"

[9] "CP256.2189_7.39" "CP256.2188_8.01" "CP256.2188_12.04" "CP256.2192_12.81"

[13] "CP256.2195_9.35" "CP257.2208_8.61" "CP257.2212_8.49" "CP257.2211_8.76"

[17] "CP257.2230_9.35" "CP258.2346_10.85" "CP284.2441_10.46" "CP284.2446_12.11"

[21] "CP284.2446_12.34" "CP284.2456_12.98" "CP285.2479_12.20" "CP291.2169_11.78"

[25] "CP318.2395_11.93" "CP319.2431_12.83" "CP324.2337_12.76" "CP337.2700_12.81"

[29] "CP338.2822_12.02" "CP338.2837_11.78" "CP352.2607_9.40" "CP354.2813_9.64"

[33] "CP355.2892_9.58" "CP358.3328_12.80" "CP368.2532_11.52" "CP388.2754_8.83"

[37] "CP390.2308_11.43" "CP410.2538_8.81" "CP496.2542_10.14" "CP496.3411_10.04"

[41] "CP496.3401_11.24" "CP496.3426_10.18"

72 clusters of isotopic peaks (M+1, M+2) were detected and only the monoisotopic peak was retained for biological investigations

**mz_RT peak isotopic peaks**

CP221.1162_11.87 CP222.1204_11.90/

CP233.1561_11.07 CP234.1595_11.09/

CP235.1680_11.85 CP236.1728_11.67/CP237.1846_11.76

CP237.1370_12.09 CP238.1415_12.27/

CP256.2180_9.48 CP257.2212_9.50/CP258.2248_9.57

CP256.2181_8.58 CP257.2226_8.69/

CP256.2192_10.00 CP257.2225_9.99/

CP256.2610_9.57 CP257.2644_9.57/CP258.2690_9.73

CP256.2621_9.72 CP257.2656_9.73/CP258.2702_9.88

CP256.2624_8.77 CP257.2651_8.86/

CP284.2958_13.07 CP285.2997_13.08/CP286.3024_12.92

CP307.2654_12.69 CP308.2671_12.67/

CP308.2417_11.66 CP309.2448_11.61/

CP308.2958_12.60 CP309.2987_12.65/

CP324.2887_12.31 CP325.2920_12.34/

CP324.2960_12.28 CP325.3000_12.35/

CP346.2120_12.29 CP347.2149_12.25/

CP348.2266_11.65 CP349.2292_11.65/

CP348.2888_12.15 CP349.2922_12.16/

CP354.2845_10.47 CP355.2885_10.32/CP356.2903_10.24

CP355.2628_8.49 CP356.2653_8.46/

CP355.2643_7.59 CP356.2676_7.53/

CP372.3476_11.61 CP373.3502_11.59/

CP373.2737_8.42 CP374.2763_8.42/

CP373.2750_7.49 CP374.2772_7.50/

CP376.2539_12.91 CP377.2568_12.95/

CP376.3202_13.10 CP377.3227_13.07/

CP380.3713_12.52 CP381.3741_12.34/

CP380.3720_0.69 CP381.3755_0.78/

CP380.3722_11.29 CP381.3752_11.30/

CP380.3723_12.73 CP381.3754_12.76/

CP388.2752_9.19 CP389.2781_9.27/

CP388.3404_9.27 CP389.3466_9.39/

CP390.2989_13.73 CP391.3005_13.75/

CP390.3001_12.86 CP391.3006_12.81/

CP394.3279_11.14 CP395.3319_11.14/

CP394.3279_12.57 CP395.3331_12.63/

CP410.3238_9.33 CP411.3298_9.42/

CP412.2866_6.38 CP413.2890_6.33/

CP412.2842_7.25 CP413.2873_7.22/

CP414.3005_8.54 CP415.3044_8.43/

CP414.3018_8.81 CP415.3032_8.79/

CP414.3018_7.90 CP415.3046_7.87/

CP414.3019_8.11 CP415.3039_8.11/

CP425.2166_12.37 CP426.2195_12.36/

CP426.3233_7.44 CP427.3256_7.39/

CP430.2950_7.38 CP431.2978_7.25/

CP430.2964_6.28 CP431.3001_6.33/

CP432.3105_8.58 CP433.3141_8.72/

CP432.3126_7.88 CP433.3154_7.87/

CP432.3102_8.84 CP433.3141_8.72/

CP432.3131_7.54 CP433.3154_7.55/

CP436.4354_0.77 CP437.4378_0.76/

CP448.3048_7.27 CP449.3083_7.23/

CP448.3075_6.31 CP449.3097_6.29/CP450.3230_6.48

CP450.3225_7.91 CP451.3261_7.89/CP452.3289_7.88

CP450.3226_8.79 CP451.3243_8.84/

CP450.3213_8.07 CP451.3267_8.06/CP452.3289_7.88

CP462.2691_5.25 CP463.2706_5.18/

CP464.2860_5.81 CP465.2872_5.91/

CP466.3155_7.31 CP467.3188_7.24/

CP466.3174_6.32 CP467.3207_6.34/CP468.3227_6.34

CP466.3151_6.77 CP467.3229_6.66/

CP500.3057_6.00 CP501.3094_6.05/

CP669.5567_12.45 CP670.5598_12.43/

CP669.5571_12.55 CP670.5600_12.54/

CP868.3673_4.75 CP869.3700_4.77/

CP868.3689_3.95 CP869.3745_3.84/

CP899.6345_8.78 CP900.6395_8.78/

CP921.6201_7.88 CP922.6245_7.89/

CP931.6240_7.29 CP932.6268_7.20/

CP948.6482_7.21 CP949.6532_7.22/

**CN data**

7 peaks were removed after sva batch correction

[1] "CN134.8000_13.19" "CN134.8000_14.20" "CN233.1000_12.25" "CN234.1000_12.18"

[5] "CN323.1000_9.24" "CN323.1000_9.89" "CN385.2993_15.58"

No isotopic peaks were removed for this set of data, largely because the correlation between peaks within the isotopic peak cluster was not high enough to meet the criteria. For example, CN408.2000_8.71/CN409.2000_8.72 seems to be a pair of isotopic peaks, but the correlation of the peak intensities is only 0.49.

Appendix 4 Summary of accurate mass measurements of CP LC-MS and MS^2^ analysis of serum from the clinical cows.

| **[M+H]^+^**  ***m/z*** | **LC-MS**  **RT (min)** | **[M+H]^+^**  ***m/z*** | **LC-MS/MS**  **RT (min)** | **MS^2^ product ions**  **(abundance) (loss (amu))** | **Elemental composition of the parent ion** | **Error**  **(ppm)** | **Proposed metabolite identity** |
| --- | --- | --- | --- | --- | --- | --- | --- |
| 337.2529 | 6.31 | 337.25287 | 9.15 | 319.24057 (100%) (-18);  209.13165 (30%) (-128);  227.14209 (15%)(-110);  255.17326 (5%) (-82) | C_24_H_32_O | 0.824 | Unknown |
| 337.2540 | 6.53 | 337.25215 | 10.26 | 319.24057 (-100%) (-18), 295.20441 (5%) (-42),  255.17326 (5%) (-82),  227.14209 (20%) (-110), 209.13164 (30%) (-128) | C_24_H_32_O | 4.174 | Unknown |
| 412.2842 | 7.25 | 412.28617 | 9.15 | 337.25126 (100%) (-57);  319.24081 (40%) (-18);  394.27252 (5%)(-18) | C_26_H_38_O_3_N | -1.02 | Glycocholic acid (minus 3 H_2_O) |
| 414.2997 | 8.99 | 414.30113 | 10.62 | 339.26704 (100%) (-75);  396.28846 (10%) (-18);  321.25657 (50%) (-93);  243.19294 (10%) (-171);  229.17335 (10%); (-185);  215.17716 (10%) (-199);  203.15636 (8%) (-211); 278.17416 (8%) (-136); 304.18975 (8%)(-110);  158.08091 (5%) (-256); | C_26_H_40_O_3_N | 2.074 | Glycochenodeoxycholic acid  (minus 2 H_2_O) |
| 414.3018 | 8.81 | 414.30013 | 10.40 | 339.26663 (100%) (-75), 321.25616 (40%) (-93), 396.28793 (10%) (-18); 215.17857 (10%) (-199);  201.16306 (8%) (-213);  238.14275 (8%)(-176);  252.15826 (5%)(-162);  278.17380 (5%)(-136);  304.18930 (5%)(-106);  158.08073 (5%)(-256) | C_26_H_40_O_3_N | -0.339 | Glycochenodeoxycholic acid  (minus 2 H_2_O)  (possible hydroxyl isomer of 414.29970) |
| 430.2950 | 7.38 | 430.28617 | 9.15 | 412.28302 (100%) (-18);  337.25148 (25%) (-93);  319.24093 (10%) (-111) | C_26_H_39_O_4_N | -0.43 | Glycocholic acid (minus 2 H_2_O) |
| 432.3102 | 8.84 | 432.31054 | 10.62 | 414.29910 (100%) (-18);  339.26773 (5%) (-93) | C_26_H_42_O_4_N | -0.683 | Glycochenodeoxycholic acid  (minus 1 H_2_O) |
| 432.3105 | 8.58 | 432.30972 | 10.40 | 414.29867 (100%) (-18);  339.26699 (4%) (-93) | C_26_H_42_O_4_N | -2.580 | Glycochenodeoxycholic acid  (minus 1 H_2_O)  (possible hydroxyl isomer of 432.31054) |
| 448.3048 | 7.27 | 448.30625 | 9.15 | 412.28342 (100%) (-36);  430.29377 (95%) (-18);  337.25185 (15%) (-111);  319.24137 (5%) (-129) | C_26_H_41_O_5_N | -2.119 | Glycocholic acid (minus 1 H_2_O) |
| 450.3209 | 8.52 | 450.32011 | 10.40 | 432.30872 (100%) (-18);  414.29825 (40%) (-36) | C_26_H_44_O_5_N | -3.546 | Glycochenodeoxycholic acid |
| 450.3226 | 8.79 | 450.32096 | 10.62 | 414.29913 (100%) (-36);  432.31009 (40%) (-18);  339.26782 (3-5%) (-111);  357.27844 (3-5%) (-93) | C_26_H_44_O_5_N | 0.229 | Glycochenodeoxycholic acid  (possible hydroxyl isomer of 450.32011) |
| 464.2832 | 7.29 | 464.28152 | 10.89 | 339.26670 (100%) (-125); 321.25624 (40%) (-143); 382.20298 (20%) (-82); 354.17178 (15%) (-110); 302.14073 (10%) (-162); 229.15784 (10%) (-235); 215.17861 (10%) (-249); 368.18733 (10%) (-96); 328.15623 (15%) (-136); 446.27033 (5%) (-18); 203.14236 (5%) (-261); 257.18898 (4-5%) (-207); 288.12519 (4-5%) (-176); 208.06305 (5%) (-256); | C_30_H_34_N_5_ C_18_H_38_O_7_N_7_  C_22_H_38_O_2_N_7_S  C_26_H_42_O_4_NS  C_22_H_46_ON_3_S_3_  C_29_H_38_O_4_N C_19_H_42_O_2_N_7_S_2_ | 1.394  -2.591  2.799  -2.985  3.809  4.275  -4.462 | Unknown  (possible positional isomer of 464.3057) |
| 464.2844 | 5.97 | 464.29910 | 8.53 | 446.28802 (100%) (-18); 428.27751 (20%) (-36); 410.26707 (10%) (-54); 335.23522 (~5%) (-129); 535.24573. (~5%) (+70) | C_26_H_42_O_6_N | -3.37 | Glycocholic acid |
| 464.3057 | 6.15 | 464.28235 | 10.40 | 339.26673 (100%) (-125);  321.25628 (40%) (-143);  382.20308 (20%) (-82);  208.06306 (15%) (-256);  288.12520 (15%) (-146);  354.17181 (15%) (-110);  254.17181 (10%) (-210);  268.18747 (8%) (-196);  302.14077 (8%) (-162);  215.17867 (8%) (-249);  201.16312 (15%) (-263);  243.20968 (8%) (-221);  446.27017 (5%) (-18);  257.18896 (5%) (-207);  229.15788 (5%) (-235) ;  157.14759 (5%) (-307);  276.12521 (5%) (-188) | C_18_H_38_O_7_N_7_ C_26_H_42_O_4_NS  C_19_H_42_O_2_N_7_S_2_  C_30_H_34_N_5_  C_27_H_38_N_5_S C_22_H_38_O_2_N_7_S | -0.803  -1.197  -2.674  3.182  -4.078  4.586 | Unknown  (possible positional isomer of 464.2832) |
| 466.3166 | 7.60 | 466.31739 | 9.15 | 430.29407 (100%) (-36); 412.28338 (85%) (-54); 448.30464 (40%) (-18); 337.25194 (10%) (-129); 355.26244 (4%) (-111); 373.27326 (3%) (-93) | C_26_H_43_NO_6_ | 0.612 | Glycocholic acid |
| 472.3031 | 8.78 | 472.30255 | 10.62 | 454.29074 (100%) (-18);  397.26953 (80%) (-75); | C_24_H_38_O_3_N_7_ C_23_H_42_O_7_N_3_ | .-1.089 1.742 | Unknown |
| 472.3039 | 7.66 | 472.30177 | 10.35 | 454.29050 (100%) (-18);  397.26932 (30%) (-75);  396.28526 (5%) (-76) | C_24_H_38_O_3_N_7_ C_23_H_42_O_7_N_3_ | 0.091 -2.741 | Unknown |
| 488.2975 | 7.19 | 488.29848 | 9.11 | 470.28532 (100%) (-18), 452.27529 (10%) (-36), 413.26427 (80%) (-75) | C_24_H_38_O_4_N_7_ | 1.026 | Unknown |
| 498.2874 | 6.45 | 498.28852 | 8.93 | 462.26558 (100%) (-36); 480.27590 (70%) (-18) | C_26_H_44_NO_6_S | -4.105 | Unknown |
| 500.3039 | 7.45 | 500.30223 | 10.89 | 464.28088 (100%) (-36); 482.29153 (40%) (-18); | C_26_H_46_NO_6_S | -0.27 | Taurochenodeoxycholic acid |
| 516.2988 | 6.45 | 516.29890 | 8.93 | 498.28625 (100%) (-18); 480.27571 (70%) (-36); 462.26520 (60%) (-54); | C_26_H_46_NO_7_S | -0.29 | Taurocholic acid |
| 533.32750 | 5.20 | 533.32548 | 8.93 | 516.29672 (-100%) (17), 498.28634 (65%) (-35), 480.27570 (-20%) (53), 462.26510 (-5%) (71) | C_18_H_45_O_10_N_8_ | 0.308 | Unknown |
