# Supplementary material for: Serum metabolomics using ultra performance liquid chromatography coupled to mass spectrometry in lactating dairy cows following a single dose of sporidesmin
Source: Metabolomics. 2018 Apr 17;14(5):61. doi: 10.1007/s11306-018-1358-4 (PMC5904237; doi:10.1007/s11306-018-1358-4)
Supplement: Supplementary file 2 — Supplementary material 2 (DOCX 26 KB) [file 11306_2018_1358_MOESM2_ESM.docx]

# Supplementary file: Experimental extended

# Journal: Metabolomics

# Serum metabolomics using ultra performance liquid chromatography coupled to mass spectrometry in lactating dairy cows following a single dose of sporidesmin

## Zoe M Matthews^1^, Patrick J B Edwards^1^, Ariane Kahnt^2^, Mark G Collett^1^, Jonathan C Marshall ^1^, Ashton C Partridge^2^, Scott J Harrison^2^, Karl Fraser^3^, Mingshu Cao^3,^ Peter J Derrick^2*^

^1^ Massey University, Palmerston North, New Zealand; ^2^ University of Auckland, Auckland, New Zealand;
^3^ AgResearch Grasslands, Palmerston North, New Zealand, * Deceased.

^§^ Authors for correspondence: [z.matthews@massey.ac.nz](mailto:z.matthews@massey.ac.nz)
 [p.j.edwards@massey.ac.nz](mailto:p.j.edwards@massey.ac.nz)

### 2.2 Liquid chromatography - mass spectrometry

The samples were cooled in the autosampler at 4 °C, and 2 μL of each was injected on the column (25 °C), using a flow rate of 400 μL/min, and the gradient elution programme as described in Fraser *et al.*(2013).

The mobile phases consisted of an aqueous 0.1 % formic acid solution (solvent A) and an acetonitrile:formic acid (99.9:0.1, *v:v*) mixture (solvent B). Solvent B was held at 5% from 0–0.5 min, 5–99% from 0.5 to 13 min, 99% from 13 to 15 min, and returned to 5 % from 15 to 16 min and allowed to equilibrate for a further 4 min prior to the next injection. Mass spectral data were collected using a mass range of *m/z* 60–1200, a resolving power of 25,000 and a maximum trap fill-time of 100 ms. The samples were run in both positive and negative ionization modes.

### 2.3 Tandem mass spectrometry coupled to liquid chromatography

The mobile phases were water with 0.1 % formic acid (A), and acetonitrile with 0.1 % formic acid (B). A 20 min gradient was applied as follows: (B) was kept at 5 % for 1 min, increased to 95 % up to 12 min, and kept at this ratio for 3 min. The reconditioning of the column back to 5 % (B) occurred over 5 min. The sample was injected using a volume of 1 μL at a flow rate of 350 μL/min. The eluent from the first 0.5 min was diverted to waste.

The experimental conditions were: sheath gas flow (N_2_), 42 arbitrary units; auxillary gas flow (N_2_), 12 arbitrary units; sweep gas (N_2_), 1 arbitrary unit; ion source voltage 3.5 kV (positive mode) or -2.5 kV (negative mode); and ion transfer tube temperature 338 °C. The mass spectrometer was operated in both full scan mode and data-dependent MS^2^ fragmentation mode for the pooled sample. The *m/z* range between 150-2000 was acquired in the full scan mode using the ion trap with an S-lens RF level of 60 %, and a maximum injection time of 100 ms. An isolation width of 1 *m/z* unit was chosen for the quadrupole mass analyser in the targeted MS^2^ scan mode, and the most intensive 20 ions were subjected to collision-induced dissociation (CID). Normalized collision energy of 35 % was applied, and the formed product ions were detected by the Orbitrap, the resolution of which was set to 30,000, and the scan range was *m/z* 50-1000.

### 2.4 Data processing and statistical analysis

### Generalised additive model (GAM) calculations:

The model fitted was as follows:

$$\log(X_{t})=\log(X_{0})+ f_{G}(t)+e_{t}$$

where X*_t_* was the variable at time *t*, *X_0_* was the variable at time 0, *f_G_(t)* is a smooth curve for each group, and *e_t_* is the residual, distributed normally. The mixed GAM computational model (mgcv) package (Wood, 2006, 2011) within the Rstatistics environment (R Development core team (2012)) was used to fit this model, with the smoothness of the function *f_G_* estimated by cross-validation. To assess whether there was a difference between groups, this model was compared to one where *f_G_(t) = f(t)* for each group *G*, i.e. a model where the change through time was similar for all groups. Model comparison was done by computing the Akaike Information Criterion (AIC) for each model. The model with the lowest AIC was deemed the most appropriate.

### Time series calculations:

Time series analysis was performed using in-house routines written for RStudio (Version 0.97.449, RStudio, Boston, MA, USA). Peak (*m/z*_RT) intensity versus time curves were plotted for each cow. The m/z_RT peaks that differed the most between groups were identified by ranking using *p*-values and shrinkage discriminant analysis (SDA). This was done as follows:

The difference between each cow at each time point was computed using mean squared difference between the measurements of the $i-th cow$and$j-th cow$:

$$d_{ij} = 1/T\sum_{1}^{T} \left( {cow}_{it}-{cow}_{jt} \right){}^{2}$$

Where T is the number of time points.

The total variation within a group (sum of squares) was then calculated. This was given by:

$$\mathrm{group}_{\mathrm{variation}}=\frac{1}{2n}\sum_{i,j} d_{ij}^{2}$$

The within-group variation was then the sum of all the total variation across each group:

$$\text{Within}_{\text{variation}}=\sum_{groups} \text{group}_{\text{variation}}$$

The between-group variation was then computed as the difference between the total variation in the dataset (irrespective of the group), minus the within-group variation:

$$\text{Between}_{\text{variation}}=\text{Tota}\text{l}_{\text{variation}}-\text{ W}\text{ithin}_{\text{variation}}$$

An F-statistic was then computed. This was given by:

$$F=\frac{(\frac{\text{B}\text{etween}_{\text{variation}}}{g-1)}}{(\frac{\text{Within}_{\text{variation}}}{n-g)}}$$

Where *g* denotes group and *n* is sample size. To allow the ranking of peaks based on their F-values, we first assessed the likelihood of the F-value arising by chance via a permutation test. For each peak, group labels were permuted 10,000 times and F-values recomputed for each permutation. The *p*-value was defined as the proportion of permutations that yielded a larger F-value than that computed from the true data. These *p*-values were not corrected for multiple testing, thus a *p*-value <0.05 was not regarded as statistically significant. However, ranking by *p*-value did allow the identification of peaks that were more likely to separate the groups. A small *p*-value suggested that the results identified from the data were unlikely to have arisen by chance. The data were ranked by *p*-value, as well as by SDA ranking. The SDA ranking determined a ranking of predictors by computing correlation-adjusted T-scores (CAT) between the group centroids and the pooled mean. These ranks were combined to produce a new rank. This was done using:

Newrank = SDA2rank + P2rank

The data were then plotted in the order of this new rank.
